# Exploring the influencing factors of academic doctoral students’ academic innovation behavior in the context of generative artificial intelligence: Self-determination theory and motivation-opportunity-ability perspectives

**DOI:** 10.1371/journal.pone.0355186

**Published:** 2026-08-06

**Authors:** Yanqin Liu, Miao Zhang, Yuwen Peng

**Affiliations:** 1 School of Political Science and Public Administration, Wuhan University, Wuhan, Hubei, China; 2 Graduate School, Xizang University, Lhasa, Xizang Autonomous Region, China; Golestan University, IRAN, ISLAMIC REPUBLIC OF

## Abstract

**Purpose:**

Although generative artificial intelligence (GenAI) has increasingly reshaped academic research practices, existing studies have mainly examined its technical affordances or isolated factors such as AI literacy. Less attention has been paid to how different types of academic motivation are translated into doctoral students’ academic innovation behavior under varying opportunity and ability conditions. Drawing on Self-Determination Theory and the Motivation-Opportunity-Ability framework, this study examines the effects of autonomous motivation, controlled motivation, and amotivation on academic doctoral students’ academic innovation behavior, and further investigates the moderating roles of resource conditions, encouragement and support, creative self-efficacy, and AI literacy.

**Design/methodology/approach:**

Based on survey data from 502 academic doctoral students, this study tested the direct effects of autonomous motivation, controlled motivation, and amotivation on academic innovation behavior. It also examined whether resource conditions, encouragement and support, creative self-efficacy, and AI literacy moderated the relationships between motivation and academic innovation behavior. Data were analyzed using SPSS 27.0, AMOS 26.0, and PROCESS 4.0.

**Findings:**

Autonomous motivation and controlled motivation positively predict academic innovation behavior, whereas amotivation negatively predicts such behavior. Resource conditions, encouragement and support, creative self-efficacy, and AI literacy significantly moderate these relationships. Specifically, these opportunity and ability factors strengthen the positive effects of autonomous and controlled motivation on academic innovation behavior and buffer the negative effect of amotivation.

**Research implications:**

Theoretically, this paper complements the integrated application of SDT and MOA in doctoral education research and enriches the multi-factor moderating mechanism of academic innovation under GenAI context. Practically, universities need to build supportive academic environments that combine motivational support, adequate research resources, responsible GenAI training, and the cultivation of creative self-efficacy.

**Originality:**

It clarifies how motivation, opportunity, and ability jointly shape academic doctoral students’ academic innovation behavior, thereby providing a more nuanced explanation of doctoral students’ innovation in the GenAI-supported academic environment.

## Introduction

Generative artificial Intelligence (GenAI) technologies, exemplified by ChatGPT and DeepSeek, are experiencing explosive growth. Their extraordinary creativity and adaptive capabilities in core domains such as natural language processing, image generation, and data analysis have far surpassed the boundaries of traditional algorithms, becoming the primary driving force behind innovation across industries [1].In the realm of academic research, GenAI has achieved deep integration across the entire scientific process. From formulating research hypotheses and processing textual materials to interpreting data results and synthesizing linguistic logic, its powerful enabling effects have not only significantly enhanced research efficiency but are also widely regarded by the academic community as a disruptive force reshaping existing scientific paradigms [2,3].As the reserve force for national scientific and technological innovation and the backbone of future academic research, the academic innovation activities of research doctoral students serve not only as a key dimension for measuring their individual research competence and academic potential, but also as a core benchmark for evaluating the quality of doctoral education. The rise of GenAI provides powerful technical support for research doctoral students to break through research bottlenecks and solve scientific challenges, while also offering significant potential opportunities for the emergence of innovative academic thinking and the implementation of innovative practices.

However, the release of technological dividends does not inherently correspond to enhanced innovation performance. The academic application of GenAI consistently exhibits the distinct characteristics of a double-edged sword: while empowering academic innovation, it also harbors multiple challenges [4]. On the one hand, overreliance on GenAI may lead to a decline in critical thinking among doctoral students, narrow their informational horizons, and even trigger academic misconduct [5–7], further exacerbates the deep-seated conflict between instrumental rationality and academic autonomy, posing a potential erosion to the very essence of scholarly innovation. On the other hand, there exists a significant disparity in the effectiveness of GenAI application among academic doctoral students: some leverage GenAI to achieve breakthroughs in top-tier research outputs, while others remain confined to conventional academic outputs [8].This core contradiction concerns both the full realization of GenAI and the effective stimulation of academic doctoral students’ innovative behavior, demanding dual responses through theoretical elaboration and empirical verification.

Research on individual innovation behavior originated in the field of organizational behavior. West and Farr (1989, 1990) defined individual innovation behavior as the behavioral process by which individuals consciously generate, promote, and implement new ideas, methods, or processes within their work roles [9,10]. This classic framework emphasizes that innovative behavior is not a single moment of creative insight, but rather a multi-stage, intentional sequence of actions, laying the theoretical foundation for subsequent research on innovative behavior in all contexts. In recent years, researchers have begun to apply this framework to the context of doctoral education. Using a sample of doctoral students, Zhang et al.(2024) directly adopted West and Farr’s three-stage “generation–promotion–implementation” model and operationalized doctoral students’ innovative behavior as their active generation, advocacy, and implementation of novel and valuable ideas or methods during the research process [11]. This study confirmed the applicability of the classic innovation behavior framework to the doctoral student population and highlighted the distinct nature of the “research process” as the context in which such behavior occurs. Doctoral students’ innovative behavior exhibits distinctive characteristics within the academic context. Baptista et al.(2015) specifically explored the nature of the “original contribution to knowledge” in doctoral degrees, pointing out that originality is not merely reflected in the final dissertation but permeates the entire research process—including problem formulation, theoretical construction, methodological design, and data interpretation [12].This perspective suggests that doctoral students’ academic innovation should not be measured solely by the final publication but should also take into account the exploration, trial-and-error, and refinement that occur at every stage of the research process. Furthermore, GenAI is currently profoundly reshaping the nature of academic research. Drawing on a technology-availability perspective, Lu et al. (2025) empirically tested the positive impact of AI literacy on doctoral students’ innovative behavior and identified the “critical use of AI tools to support the research process” as a key component of such behavior [13]. This study adopts this new dimension to reflect the contemporary context brought about by technological change. In summary, this study defines academic doctoral students’ academic innovation behavior as the conscious engagement in the generation, promotion, and implementation of novel and valuable ideas, methods, perspectives, or outcomes during the academic research process. Specifically, this includes identifying original research questions; adopting or developing new theoretical and methodological approaches; critically using GenAI tools to support academic inquiry; and producing research outcomes that demonstrate originality, rigor, and academic value. This definition emphasizes that academic doctoral students’ academic innovation behavior is not limited to the final published outcomes but also encompasses the entire behavioral process through which they explore, refine, and implement innovative academic ideas.

Existing research has preliminarily examined the factors influencing doctoral students’ innovative behavior and confirmed that GenAI literacy exerts a positive effect on such behavior [13]. However, it must be clarified that Academic innovation behavior is not a routine academic task, but a highly self-regulated, uncertain, and cognitively demanding process that requires doctoral students to generate, refine, promote, and implement novel academic ideas [14]. Therefore, it is necessary to first examine the motivational basis of such behavior. Given the inherent complexity of this process, a single theoretical lens is insufficient to capture its full mechanism. Currently, the academic community lacks systematic exploration into the intrinsic mechanisms underlying doctoral students, particularly academic doctoral students’ academic innovation behaviors within the context of GenAI. Consequently, it remains challenging to establish a targeted academic innovation support system.

To address this research gap, this study employs empirical methods to explore the factors that generate and moderate academic innovation behaviors among research doctoral students in the context of GenAI. By integrating Self-Determination Theory (SDT) with the Motivation-Opportunity-Ability (MOA) framework, this research aims to comprehensively analyze the key factors influencing academic innovation behaviors among research doctoral students. On the one hand, SDT provides a foundational lens for distinguishing different qualities of motivation—autonomous, controlled, and amotivation—thereby explaining the internal drive that makes doctoral students willing to invest effort in academic innovation. motivation alone cannot fully explain whether doctoral students actually transform innovative intentions into concrete academic practices. In the GenAI-supported research environment, academic innovation also depends on whether doctoral students have sufficient external opportunities and individual abilities [15]. The MOA framework complements SDT by explaining how opportunity-related factors, such as resource conditions and encouragement and support, and ability-related factors, such as creative self-efficacy and AI literacy, shape the process through which motivation is translated into academic innovation behavior. Thus, SDT explains the type of doctoral students’ motivation, whereas the MOA framework explains the boundary conditions under which such motivation can be converted into academic innovation behavior.

## Literature review and research hypotheses

### Academic innovation behavior of doctoral students: A review of empirical studies

As the main force in knowledge production and a reserve of future scientific talent, doctoral students’ academic innovation activities are attracting increasing attention from the academic community. Some empirical studies have begun to explore the factors that promote or hinder doctoral students’ academic innovation, but most of these studies remain scattered.

On the one hand, exploring the impact of organizational support. Zhang et al. (2025) found that paradoxical leadership—characterized by a balance between structure and flexibility—significantly predicted graduate students’ creative behavior, with creative role identity and psychological safety acting as parallel mediators [16]. Wei et al. (2026) further demonstrated that supervisors’ support for innovation significantly and positively predicts graduate students’ proactive innovative behavior, with innovation efficacy and engagement in the creative process playing a chained mediating role [17]. Based on the Affective Event Theory, Zhang et al.(2024) demonstrated that advisor support mediates the relationship between challenging time pressure and obstructive time pressure on graduate students’ innovative behavior, both enhancing the positive effects of challenging time pressure and mitigating the negative effects of obstructive time pressure [11]. Li et al. (2025)further explored how research-related stress among graduate students is transformed into motivation and innovative behavior, revealing that support from advisors, family, or teams directly promotes innovative behavior among graduate students, with intrinsic motivation and role identification serving as sequential mediators [18].On the other hand, examining the influence of individual abilities and psychology. A study by Han et al.(2022) showed that creative self-efficacy significantly moderates the relationship between academic support and innovative behavior in research among graduate students [19].Zhang et al.(2025) investigated how graduate students’ orientation types influence their research innovation behavior and found that promotion-oriented graduate students exhibited stronger self-regulation, leading to higher levels of research innovation, while prevention-oriented students experienced severe self-regulation fatigue, which hindered their innovative potential [20]. In the context of GenAI, recent research has increasingly emphasized the role of artificial intelligence literacy and AI support in learning environments, and is gradually being applied to explore student innovative behavior. Lu et al.(2025) examined the impact of AI literacy on doctoral students’ innovative behavior and found that AI literacy has a significant positive effect on innovative behavior, while AI competence and affective engagement play a chained mediating role in the relationship between AI literacy and innovative behavior [13]. Building on SDT, He et al.(2026) investigated how knowledge of artificial intelligence influences doctoral students’ innovative behavior. They demonstrated that AI dependence mediates the relationship between AI knowledge and doctoral students’ innovative behavior, and that achievement motivation weakens the negative relationship between AI dependence and doctoral students’ innovative behavior [21].

**Fig 1:**
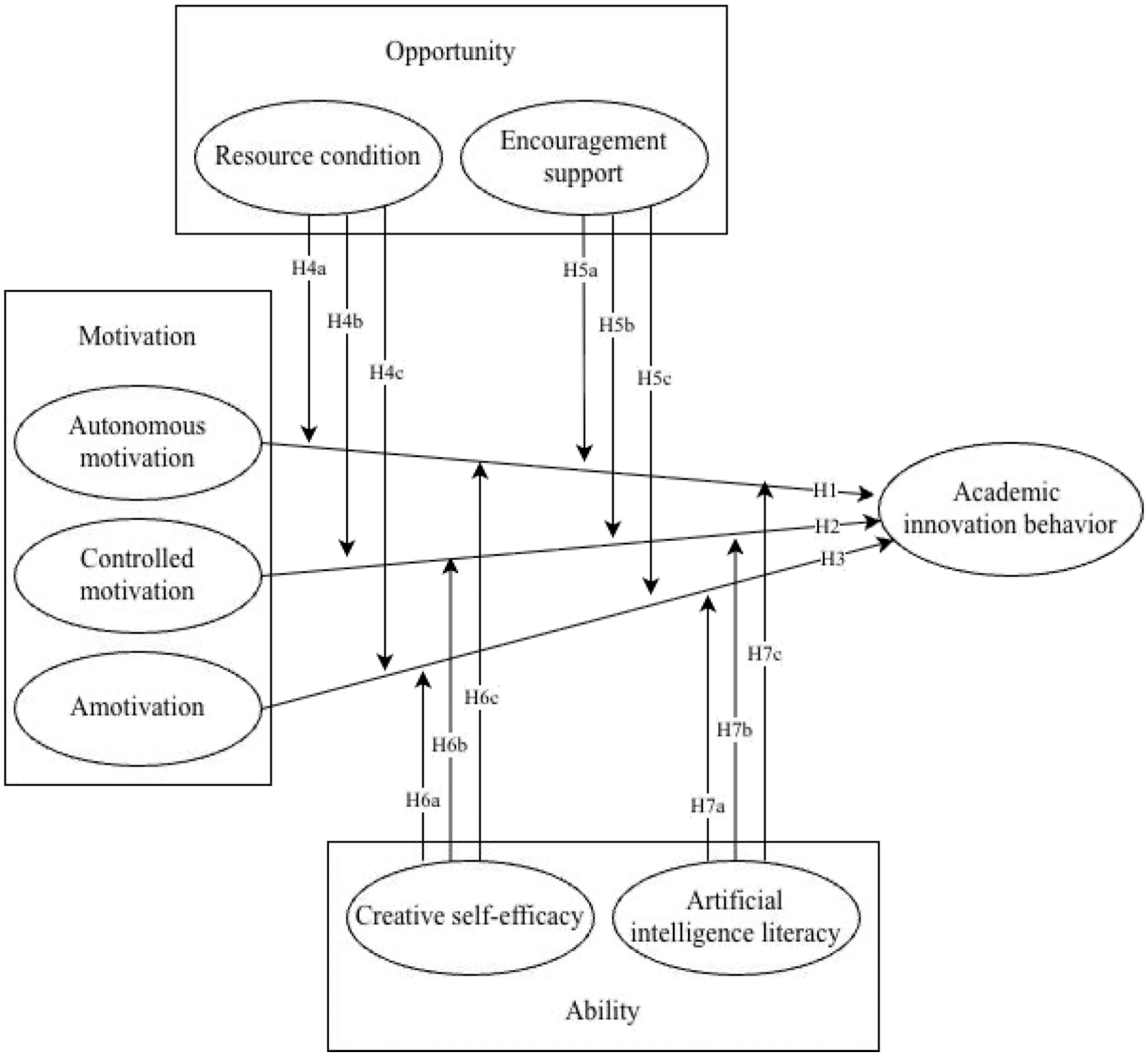
Research hypothesis framework.

In sum, prior studies have established that doctoral students’ academic innovation behavior is shaped by a constellation of factors spanning supervisory support, institutional resources, individual motivation, self-efficacy, and AI knowledge. However, the existing research lacks a comprehensive and integrative framework that systematically examines the interactive mechanisms through which these diverse dimensions jointly influence innovation. To address these gaps, the present study proposes an integrated model (Fig 1) grounded in SDT and the MOA framework. In our integrated model, SDT and MOA framework are not parallel constructs but rather intertwined levels. Motivation within SDT serves as an catalyst, and its influence on academic innovation behavior is shaped by the various dimensions of MOA framework. Specifically, opportunity-related factors (resource conditions and encouragement and support) act as situational support, amplifying or attenuating the effects of motivation. At the same time, ability-related factors (creative self-efficacy and AI literacy), acting as personal empowerment factors, determine whether doctoral students possess the necessary skills to translate their motivational intentions into concrete academic practices. Thus, SDT explains why doctoral students choose to innovate, while MOA clarifies the favorable conditions under which they can truly succeed in innovating.This comprehensive approach enables us to gain a more detailed and comprehensive understanding of the multifaceted determinants of innovative behavior among doctoral students in the contemporary GenAI research field. Therefore, this study is guided by the following overall research questions:

RQ1: How do the three types of motivation posited by SDT—autonomous motivation, controlled motivation, and amotivation—differentially influence the academic innovation behavior of doctoral students?

RQ2: To what extent do contextual opportunity factors, specifically resource conditions and encouragement and support, moderate the relationships between different types of motivation and academic innovation behavior?

RQ3: To what extent do individual capability factors, specifically creative self-efficacy and AI literacy, moderate the relationships between different types of motivation and academic innovation behavior?

### Self-Determination Theory (SDT)

Self-determination theory is a comprehensive framework for understanding motivational needs and psychological motivation, which can be used to explain why and how certain behavior occurs [22].This theory categorizes motivation into three main types: autonomous motivation, controlled motivation, and amotivation. Autonomous motivation refers to an individual’s engagement in behavior that is voluntary, chosen, and driven by a sense of will [23]. Gagné and Deci (2005) further defined intrinsic motivation as “an individual’s engagement in an activity because they find it important, valuable, or interesting, without being compelled by internal or external forces.” [24]. The emergence and maintenance of this motivational state depend on the fulfillment of three basic psychological needs: autonomy, competence, and relatedness [25]. When applying this theoretical framework to the academic context of doctoral students, existing research indicates that their intrinsic motivation manifests as the attribution of personal value to academic research activities, stemming from a genuine interest in the research itself and a sense of intrinsic satisfaction [26].

Specifically, this is reflected in interest-driven academic exploration, a deep identification with academic values, and voluntary academic engagement. In this study, autonomous motivation is a state of internal motivation in which doctoral students actively engage in research and learning based on intrinsic academic interest and value alignment, grounded in autonomy, competence, and the fulfillment of relational needs, and not driven by controlled factors such as external pressure or internal anxiety. Jiang et al.(2023) found that individual motivation positively promotes innovation performance, in which innovative behavior plays an intermediary role [27]. This finding indirectly confirmed the positive role of intrinsic motivation in innovative behavior. Research has further shown that students’ autonomous motivation for innovation serves as a positive predictor of their behavior [28,29]. From this perspective, doctoral students pursuing academic degrees who are motivated by autonomy in academic innovation will perceive academic innovation itself as inherently interesting and rewarding, thereby being more inclined toward academic innovation. Based on this, we propose the following hypothesis:

H1: Academic doctoral students’ autonomous motivation positively influences academic innovation behavior.

Unlike autonomous motivation, controlled motivation is primarily driven by external obligations and pressures, such as engaging in certain behaviors to alleviate feelings of guilt or shame [30].According to SDT, controlled motivation depends on an individual’s perception of the connection between behavior and expected outcomes (such as tangible rewards) [24,31]. In the academic context of doctoral students, controlled motivation manifests as viewing academic activities as a means to achieve external goals [26]. Specifically, it is characterized by engagement in academic work driven by external factors—such as degree requirements and publication pressures—or by internal pressures—such as avoiding guilt or maintaining self-esteem—and is accompanied by a lack of a sense of agency and choice. In this study, controlled motivation is a state of doctoral students engage in academic activities due to external rewards and punishments or internal coercive pressures (such as guilt or shame). The core experiences associated with this motivation are a sense of pressure, a sense of obligation, and a sense of being controlled, and the rationale for their behavior stems from external factors separate from the academic activities themselves, distinguishing it from autonomous motivation based on interest or value alignment. Research has confirmed that the more individuals are motivated by external rewards, institutional rules, or others’ expectations, the more likely their behavior is to be driven by extrinsic motivation [32]. At the same time, existing research also indicates that institutional external incentives can significantly boost students’ enthusiasm for participating in innovation activities [33].Therefore, even if students themselves lack an intrinsic interest in academic innovation, their innovative behaviors may still occur under the influence of controlled motivation triggered by external factors. Based on this, we propose the following hypothesis:

H2: Academic doctoral students’ controlled motivation positively influences academic innovation behavior.

According to self-determination theory, individuals lose motivation to engage in specific behaviors when those behaviors lack intentionality and value [34]. When applying this theoretical framework to the academic context of doctoral students, existing research has shown that amotivated doctoral students exhibit a lack of perception of the connection between behavior and outcomes in their academic activities [26]. In this study, amotivation is a state of lack of motivation exhibited by doctoral students due to their inability to perceive the connection between academic behavior and output, a lack of value or interest in academic activities, or a perceived lack of ability. The core experiences of this state include a lack of intentionality, a sense of helplessness, a sense of detachment, and low effort investment. Given this, if students themselves lack the willingness or motivation to engage in academic innovation, it will directly constrain their academic innovation behavior. Based on this, we propose the following hypothesis:

H3: Academic doctoral students’ amotivation has a negative influence on academic innovation behavior.

However, while SDT effectively explains what drives doctoral students to innovate, it is less equipped to explain when and under what conditions these motivational states are most likely to translate into concrete innovative actions. In traditional application, SDT often implies a relatively direct pathway from motivation to behavior, overlooking the critical reality that individual capabilities and external opportunities may significantly moderate this process. Therefore, to complement SDT and construct a more comprehensive model, we introduce MOA framework. MOA framework explicitly posits that behavior is not solely a function of motivation, but also depends on an individual’s ability to perform the action and the opportunity afforded by the environment. By integrating MOA framework, we can extend our investigation beyond direct effects to examine how opportunity and ability moderate the relationships proposed in H1–H3. This integration is theoretically collaborative: it allows us to go beyond the simple assumption that “motivation always leads to action” and conceptualize academic innovation as an accidental process, where the type of motivation interacts with individual abilities and environmental cues to jointly determine behavioral outcomes.

### Motivation-Opportunity-Ability (MOA)

The Motivation-Opportunity-Ability framework was initially applied to explain information processing in advertising contexts. Its core assumption posits that motivation, opportunity, and ability directly and interactively influence behavioral outcomes [35]. SDT emphasizes the role of motivation, while the MOA framework incorporates individual capabilities and external opportunities into the analytical process by introducing a situational dimension. This facilitates a deeper and more comprehensive understanding of the unfolding of individual behavior. Existing research has integrated the MOA theoretical framework with self-determination theory in the educational domain, conducting empirical analyses on the relationships among motivation, capability, opportunity, and individual behavior. These studies indicate that capability and opportunity exert a moderating influence on the process by which motivation translates into action [36–38]. Based on this research logic, this study hypothesizes that capability and opportunity will moderate the process by which motivation is transformed into academic innovation behavior.

Regarding opportunity, resource conditions and supportive encouragement are incorporated as scenario-adjusting variables, both of which can positively moderate the relationship between motivation and innovative behavior [39]. Specifically, on the one hand, as a crucial component of this dimension, resource conditions can effectively translate academic innovation motivation into tangible actions by providing the necessary tools and infrastructure [40]. Resource conditions are defined as the accessibility and adequacy of tangible and intangible assets that directly facilitate doctoral students’ research execution. In this study, this variable is specifically measured by three dimensions: (a) material infrastructure (e.g., laboratory equipment, software, and GenAI tools), (b) data and financial support (e.g., access to proprietary databases, research funding, and project budgets), and (c) platform opportunities (e.g., academic conferences and channels for publishing or displaying innovative outcomes) [41]. Existing research indicates that resource conditions, as a moderating variable, can indirectly influence students’ creativity by stimulating their motivation for innovation [42].Given this, whether doctoral students can effectively translate their academic innovation motivation into innovative practices is likely to vary significantly depending on the adequacy of available resources. On the other hand, existing research indicates that organizational support can strengthen the relationship between researchers’ intrinsic motivation and their innovative behaviors [43,44]. With the widespread adoption of GenAI in academic circles, ethical considerations and moral principles in artificial intelligence have gradually garnered significant attention [45,46]. Academic doctoral students pursuing scholarly innovation require not only verbal encouragement and support from their institutions but also the establishment of institutional safeguards that enable them to leverage GenAI for academic innovation. This approach helps prevent excessive reliance on GenAI while mitigating associated ethical and moral risks, thereby further facilitating the translation of innovative motivation into tangible practice [47]. Supportive encouragement is defined as the perceived institutional and organizational backing that validates doctoral students’ academic risk-taking. In this study, this variable is operationalized through two dimensions: (a)affirmative/verbal support(e.g., supervisory encouragement and peer recognition) [48], (b)training opportunity(e.g., GenAI-related training, standards, or guidelines) and (c) institutional safeguards (e.g., clear ethical guidelines for GenAI use, which reduce perceived risks and moral concerns while preventing over-reliance on technology), thereby enabling students to transform motivation into practice securely [49]. Based on this, we propose the following hypothesis:

H4: Resource condition significantly moderates the relationship between autonomous motivation (H4a), controlled motivation (H4b), amotivation (H4c) and academic innovation behavior.

H5: Encouragement and support significantly moderate the relationship between autonomous motivation (H5a), controlled motivation (H5b), amotivation (H5c) and academic innovation behavior.

External opportunity factors determine whether doctoral students have objective conditions to transform motivation into innovation, while individual ability factors determine whether they possess subjective capability to utilize such opportunities. This paper selects creative self-efficacy and AI literacy as two core ability moderators under GenAI background. Creative self-efficacy refers to an individual’s ability to generate and propose unique and valuable ideas [50]. Creative self-efficacy significantly enhances an individual’s confidence and creativity in tackling challenges, motivating them to adopt innovative approaches to accomplish tasks. It serves as a crucial foundational capability for driving innovation activities [51,52]. According to previous research, students with strong creative self-efficacy maintain motivation and demonstrate greater psychological resilience even when faced with setbacks and failures [53]. More crucially, existing research has identified creative self-efficacy as a key capability variable and confirmed that it plays a significant moderating role between individual motivation and innovative behavior [54]. Specifically, whether an individual’s motivation level can effectively translate into actual innovative behavior largely depends on the strength of their creative self-efficacy. Furthermore, artificial intelligence literacy is crucial for doctoral students’ scientific research. According to the definition by Lérias et al. [55], artificial intelligence literacy refers to an individual’s comprehensive ability in the cognitive understanding, practical application, technological discernment, and ethical grasp of artificial intelligence. Drawing upon but extending Lérias et al.’s framework, this study operationalizes AI literacy through four measurable dimensions: (a) cognitive understanding (knowledge of GenAI’s principles, capabilities, and limitations), (b) practical application (hands-on proficiency in using GenAI for literature review, data analysis, or draft generation), (c) technological discernment (capacity to critically evaluate GenAI-generated outputs for accuracy and relevance), and (d) ethical grasp (awareness and adherence to academic integrity and ethical guidelines when employing GenAI tools). It is worth noting that the emergence of GenAI has significantly transformed learning models and innovation approaches, requiring learners to acquire the necessary AI literacy to adapt to this shift [52]. Previous studies have further confirmed that artificial intelligence literacy exerts a significant positive influence on doctoral students’ innovative behavior [13]. AI literacy can enhance students’ confidence in effectively utilizing GenAI, thereby further stimulating their willingness to innovate and driving the implementation of innovative actions [56].It is evident that the higher the level of AI literacy, the more effectively motivation can be translated into actual innovative behavior. Based on this, we propose the following hypothesis:

H6: Creative self-efficacy significantly moderated the relationship between autonomous motivation (H6a), controlled motivation (H6b), and amotivation (H6c) and academic innovation behavior.

H7: Artificial intelligence literacy significantly moderated the relationship between autonomous motivation (H7a), controlled motivation (H7b), amotivation (H7c) and academic innovation behavior.

Based on above research hypotheses, this study proposes a conceptual model (Fig 1). In this model, autonomous motivation, controlled motivation, and amotivation exert indirect effects on academic innovation behavior. Creative self-efficacy and AI literacy moderate the relationship between autonomous motivation, controlled motivation, amotivation and academic innovation behavior. Resource conditions, encouragement and support moderate the relationship between autonomous motivation, controlled motivation, amotivation and academic innovation behavior.

## Method

### Participants and procedure

The research aimed to examined exploring the influencing factors of academic innovation behavior of academic doctoral students in the context of GenAI. To achieve this objective, we collected data through online stratified random sampling survey, with stratification based on gender, grade level, and subject type. Before data collection, we had obtained the ethical approval from Medical Ethics Review Committee of Xizang University(ZDYXLL2026029). Moreover, we provided students with detailed information about the survey’s purpose and their right to withdraw at any stage of the survey. We had also informed students via an online voice call that the survey was conducted anonymously, and no personal information will be disclosed. All participants provided verbal informed consent prior to data collection. To formally document verbal consent, researchers recorded the entire consent communication process via audio recording after notifying participants in advance. An independent research assistant who did not participate in subsequent data collection served as the witness for each verbal consent procedure. The witness signed a standardized witness record form to confirm that participants fully understood the research purpose, procedures, potential risks, voluntary participation and right to withdraw without penalty before giving verbal consent. Moreover, before filling out the questionnaire, all participants need to carefully read the relevant prompts for informed consent, and then click the “Agree to Participate” button to officially start the questionnaire survey. Only those who confirm their agreement through this button can proceed with the investigation. The verbal consent protocol were fully reviewed and formally approved by Medical Ethics Review Committee of Xizang University. After obtaining the respondent’s explicit verbal consent, the investigator assisted them in completing the online questionnaire.

The data collection took place from March 7, 2026, to March 21, 2026. Data were collected through Questionnaire Star, a questionnaire collection platform. A total of 543 questionnaires were distributed, and after excluding 41 invalid responses, 502 valid questionnaires were obtain, yielding a response rate of 92.45%. Among the valid sample, 48.8% were male and 51.2% were female. The grade of the sample was: first grade (25.9%), second grade (28.69%), third grade (24.3%), fourth grade (12.75%), and fifth grade and above (8.37%). Regarding discipline, humanities and social sciences (33.86%), science, engineering, agriculture, and medicine (36.06%), and interdisciplinary and others (30.08%). Regarding length of generative AI usage for academic research, less than 3 months(7.17%), 3–6months(10.16%), 6–12 months(37.85%) and more than 12 months(44.82%). Regarding weekly frequency of generative AI application in research, less than once a week(3.78%), 1–3 times per week(13.94%), 4–6 times per week(53.59%) and 7 times or more per week(28.69%). Table 1 shows the detailed demographic information of the sample.

**Table 1:** Sample demographic(*N* = 502).

	Number	Percentage
Gender		
Male	245	48.8%
Female	257	51.2%
Grade		
First	130	25.9%
Second	144	28.69%
Third	122	24.3%
Fourth	64	12.75%
Fifth and above	42	8.37%
Discipline		
Humanities and social sciences	170	33.86%
Science,engineering, agriculture, and medicine	181	36.06%
Interdisciplinary and others	151	30.08%
Length		
Less than 3 months	36	7.17%
3-6months	51	10.16%
6-12 months	190	37.85%
More than 12 months	225	44.82%
Frequency		
Less than once a week	19	3.78%
1-3 times per week	70	13.94%
4-6 times per week	269	53.59%
7 times or more per week	144	28.69%

### Measures

**Motivation scale** The Motivation Scale was developed by Vallerand et al. [57]. The scale consists of 10 items, including statements such as “I pursue academic innovation because I genuinely find it fascinating to explore new questions”,“I enjoy the process of proposing new ideas and solving complex problems through my research”, and “I believe that making valuable academic contributions aligns closely with my personal goals.” The scale includes three sub-dimensions: autonomous motivation(with items AM1, AM2, AM3and AM4), controlled motivation(with items CM1, CM2 and CM3), and amotivation(with items M1, M2 and M3). A 5-point Likert scale was used, ranging from 1 (strongly disagree) to 5 (strongly agree). Higher scores on the scale indicate a higher level of motivation. In overall scale demonstrated acceptable internal consistency, with Cronbach’s alpha coefficient of 0.813 for the current study. The Confirmatory factor analysis (CFA) results showed that the composite reliability (CR) was 0.84, and the average variance extracted(AVE) was 0.504, both meeting acceptable standards, indicating good convergent validity. Furthermore, the square root of the AVE was greater than correlation between the variables, providing satisfactory discriminant validity for the scale.

**Resource condition scale** The Resource Condition Scale was developed by Amabile et al. [58]. The scale consists of four items, including statements such as “I have access to the key resources needed to conduct innovative research, including data, equipment, and funding,” “I have a relatively flexible schedule that allows me to pursue exploratory research,” “I have easy access to GenAI tools,” and “I have access to platforms and opportunities for academic exchange and showcasing innovative achievements.” A 5-point Likert scale was used, ranging from 1 (strongly disagree) to 5 (strongly agree). Higher scores on the scale indicate a higher level of resource condition. In overall scale demonstrated acceptable internal consistency, with Cronbach’s alpha coefficient of 0.91 for the current study. The CFA results showed that the CR was 0.911, and the AVE was 0.72, both meeting acceptable standards, indicating good convergent validity. Furthermore, the square root of the AVE was greater than correlation between the variables, providing satisfactory discriminant validity for the scale.

**Encouragement and support scale** The Encouragement and Support Scale was developed by Amabile et al. [58]. The scale consists of four items, including statements such as “My advisor encourages me to come up with new ideas and is always willing to discuss and support them,” “My advisor supports my efforts to use new technologies, such as GenAI, to foster innovation,” and “The university provides GenAI-related training, guidelines, and guidance, which allows me to use it with greater confidence and in compliance with regulations.” A 5-point Likert scale was used, ranging from 1 (strongly disagree) to 5 (strongly agree). Higher scores on the scale indicate a higher level of encouragement and support. In overall scale demonstrated acceptable internal consistency, with Cronbach’s alpha coefficient of 0.862 for the current study. The CFA results showed that the CR was 0.832, and the AVE was 0.633 both meeting acceptable standards, indicating good convergent validity. Furthermore, the square root of the AVE was greater than correlation between the variables, providing satisfactory discriminant validity for the scale.

**Creative self-efficacy scale** The Creative Self-efficacy Scale was developed by Tierney & Farmer [50]. The scale consists of five items, including statements such as “I am confident in formulating innovative and testable research questions or hypotheses,” “I am capable of transforming a new idea into a feasible research design,” and “I can effectively search for, screen, and synthesize the literature to develop my own perspectives”. A 5-point Likert scale was used, ranging from 1 (strongly disagree) to 5 (strongly agree). Higher scores on the scale indicate a higher level of creative self-efficacy. In overall scale demonstrated acceptable internal consistency, with Cronbach’s alpha coefficient of 0.806 for the current study. The CFA results showed that the CR was 0.839 and the AVE was 0.541 both meeting acceptable standards, indicating good convergent validity. Furthermore, the square root of the AVE was greater than correlation between the variables, providing satisfactory discriminant validity for the scale.

**Artificial intelligence literacy scale** The AI Literacy Scale was developed by Wang et al. [59]. The scale consists of 12 items, including statements such as “I can tell which devices are smart and which are not,” “I know how AI technology can help me,” and “I can identify which AI technologies are used in the apps or products I use.” A 5-point Likert scale was used, ranging from 1 (strongly disagree) to 5 (strongly agree). Higher scores on the scale indicate a higher level of AI literacy. In overall scale demonstrated acceptable internal consistency, with Cronbach’s alpha coefficient of 0.943 for the current study. The CFA results showed that the CR was 0.944 and the AVE was 0.587 both meeting acceptable standards, indicating good convergent validity. Furthermore, the square root of the AVE was greater than correlation between the variables, providing satisfactory discriminant validity for the scale.

**Academic innovation behavior scale** The Academic Innovation Behavior Scale was developed by Scott & Bruce [14]. The scale includes three sub-dimensions: idea generation(with items AIB1 and AIB2), idea promotion(with items AIB3) and idea implementation(with items AIB4, AIB5 and AIB6). The scale consists of six items, including statements such as “I frequently come up with and propose new research ideas or perspectives,” “I actively seek out new methods, tools, or data to improve my research,” and “I discuss these new ideas with my advisor and peers and seek their feedback and support.” A 5-point Likert scale was used, ranging from 1 (strongly disagree) to 5 (strongly agree). Higher scores on the scale indicate a higher level of Academic innovation behavior. In overall scale demonstrated acceptable internal consistency, with Cronbach’s alpha coefficient of 0.839 for the current study. The CFA results showed that the CR was 0.869 and the AVE was 0.554 both meeting acceptable standards, indicating good convergent validity. Furthermore, the square root of the AVE was greater than correlation between the variables, providing satisfactory discriminant validity for the scale.

### Data analysis

Data analysis employed SPSS 27.0 and AMOS 26 software. Initially, CFA were utilized to establish the scale validity and the construct validity. To ensure reliability of the measurement instruments, Cronbach’s alpha coefficients were calculated for each scale. The factor analysis was determined by the Kaiser-Meyer-Olkin (KMO) measure and Bartlett’s test of sphericity. Furthermore, the Harman single-factor test was conducted to determine common method bias. Subsequently, descriptive statistics, including means, standard deviations, skewness, and kurtosis, showed the data distribution and normality. Multicollinearity was analyzed and showed to be adequate for all analysis |VIF < 5; Tolerance>0.2| [60]. Pearson’s product-moment correlation analysis was performed to examine the relationships between autonomous motivation, controlled motivation, amotivation, resource condition, encouragement and support, creative self-efficacy, AI Literacy, and academic innovation behavior. Finally, we tested whether resource condition, encouragement and support, creative self-efficacy, and AI Literacy accounted for the association between autonomous motivation, controlled motivation, amotivation and academic innovation behavior using 5000 bootstrapped samples with the PROCESS macro model 1 for SPSS [61]. Indirect effects were considered to be significant if the 95% confidence interval did not include zero [61].

## Result

### Common method bias testing

In this study, main variables were assessed using self-report questionnaires, which may have caused a common method bias. We conduct the Harman single-factor test to determine the potential impact of common method bias on the research result [62]. The results showed that 8 factors had en eigenvalue greater than 1, and the first factor accounted for 20.15%, which was less than 40% of critical standard [63]. This showed that the result had no apparent common method bias.

### Confirmatory factor analysis (CFA)

CFA was performed to evaluate the measurement model. The measurement model demonstrated good fit with data across multiple indices: χ2/df = 2.153, NFI = 0.891, CFI = 0.938, TLI = 0.932, IFI = 0.938, and RMSEA = 0.048. All fit indices met or exceed the respective recommended benchmarks, indicating that the measurement model adequately represented the data [64]. Furthermore, CR and AVE values were used to assess the convergent validity. The discriminant validity was measured by comparing square roots of AVE with inter-construct correlations. The result presented in Table 2, showed that convergent validity was confirmed as all AVE values met the 0.5 threshold, with values ranging from 0.53 to 0.778 [65].Each construct met the recommended threshold CR value of over 0.8, ranging 0.823 to 0.944 [66].Discriminant validity was confirmed as the square roots of AVE larger than a specific variable’s correlation coefficient with all other variables [67].

**Table 2:** Confirmatory factor analysis.

Construct	Item	AVE	CR	Cronbach’s alpha	Tolerance	VIF
Autonomous motivation	AM1	0.778	0.933	0.933	0.828	1.208
	AM2					
	AM3					
	AM4					
Controlled motivation	CM1	0.731	0.89	0.899	0.796	1.257
	CM2					
	CM3					
Amotivation	M1	0.681	0.865	0.864	0.789	1.267
	M2					
	M3					
Resource condition	RC1	0.72	0.911	0.910	0.718	1.393
	RC2					
	RC3					
	RC4					
Encouragement and support	ES1	0.613	0.864	0.862	0.967	1.034
	ES2					
	ES3					
	ES4					
Creative self-efficacy	CS1	0.53	0.823	0.806	0.839	1.191
	CS2					
	CS3					
	CS4					
	CS5					
AI Literacy	AIL1	0.587	0.944	0.943	0.957	1.045
	AIL2					
	AIL3					
	AIL4					
	AIL5					
	AIL6					
	AIL7					
	AIL8					
	AIL9					
	AIL10					
	AIL11					
	AIL12					
Academic innovation behavior	AIB1	0.553	0.867	0.839	–	–
	AIB2					
	AIB3					
	AIB4					
	AIB5					
	AIB6					

### Descriptive statistics and correlations

Descriptive statistics and the intercorrelations between the variables are provided in Table 3. Table 3 presents the descriptive statistics for all eight constructs examined in this study, including means, standard deviations, skewness, and kurtosis values for autonomous motivation, controlled motivation, amotivation, resource condition, encouragement and support, creative self-efficacy, AI literacy, and academic innovation behavior. First, we assessed the normality of the data distribution with skewness and kurtosis because non-normality distributed variables may result in a distorted estimation. Skewness for constructs ranged between −0.465 and 0.629, and kurtosis ranged between −1.179 and 0.625 (both within the −2 to +2 range), which suggested good distributional properties for the data [68]. Data quality was also checked, and the standardized residuals among the individual scale items ranged between 0.706 and 1.065, well below the cutoff threshold value of 3.00 [68].

**Table 3:** Descriptive statistics and correlation matrix.

Construct	1	2	3	4	5	6	7	8
1. AM		0.228***	0.272***	0.304***	0.094**	0.305***	0.071	0.385***
2. CM			0.322***	0.382***	0.136**	0.231***	0.027	0.368***
3. M				0.363***	0.049	0.224***	−0.075	−0.384***
4. RC					0.121**	0.313***	0.13**	0.338***
5. ES						0.078	0.087	−0.245***
6. CS							0.006	0.236***
7. AIL								0.054
8. AIB								
Mean	2.360	2.239	2.261	2.408	3.409	2.323	3.448	2.391
SD	0.856	0.784	0.808	0.840	1.065	0.706	0.843	0.867
Skewness	0.423	0.466	0.469	0.523	−0.465	0.214	−0.187	0.629
Kurtosis	0.121	0.273	0.457	0.405	−0.351	0.316	−1.179	0.625

*** p < 0.001, ** p < 0.01, * p < 0.05

Among the eight constructs, AI literacy demonstrated the highest mean score (M = 3.448, SD = 0.843), indicating that students generally perceived the significant importance in possessing a high level of artificial intelligence literacy in the current severe academic environment. The meanscore for encouragement and support ranks second (M = 3.409, SD = 1.065), indicating that students generally perceived the encouragement and support from their organizations in academic innovation. The mean score for control motivation is the lowest (M = 2.239, SD = 0.784), indicating that students’ motivation to engage in academic innovation activities rarely comes from external factors.

### Direct effect analyses

Table 4 presents the indirect correlation between autonomous motivation, controlled motivation, amotivation and academic innovation behavior. The result indicated that both autonomous motivation (*b* = 0.390, t = 0.385, *p* < 0.001) and controlled motivation (*b* = 0.407, t = 0.368, *p* < 0.001) exerted a significantly positive effect on academic innovation behavior. This finding suggested that individuals with higher autonomous motivation or controlled motivation possessed a stronger tendency to participate in academic innovation activities, and such motivational orientations could effectively drive the generation and practice of academic innovation behaviors. On the contrary. Conversely, amotivation (*b* = −0.411, t = −0.387, *p* < 0.001) had a significantly negative impact on academic innovation behavior, which reflected that individuals with amotivation showed obvious apathy and low initiative toward academic innovation. They not only lacked intrinsic interest in exploring academic innovation, but also had no external motivation to engage in relevant innovative practices, thus being unwilling to devote time and energy to academic innovation behavior.

**Table 4:** Results of direct effect.

Hypotheses	Paths	*b*	t	Result
H1	Autonomous motivation → academic innovation behavior	0.390	0.385	Supported
H2	Controlled motivation → academic innovation behavior	0.407	0.368	Supported
H3	Amotivation → academic innovation behavior	−0.411	−0.387	Supported

### Moderation analyses

**Fig 2:**
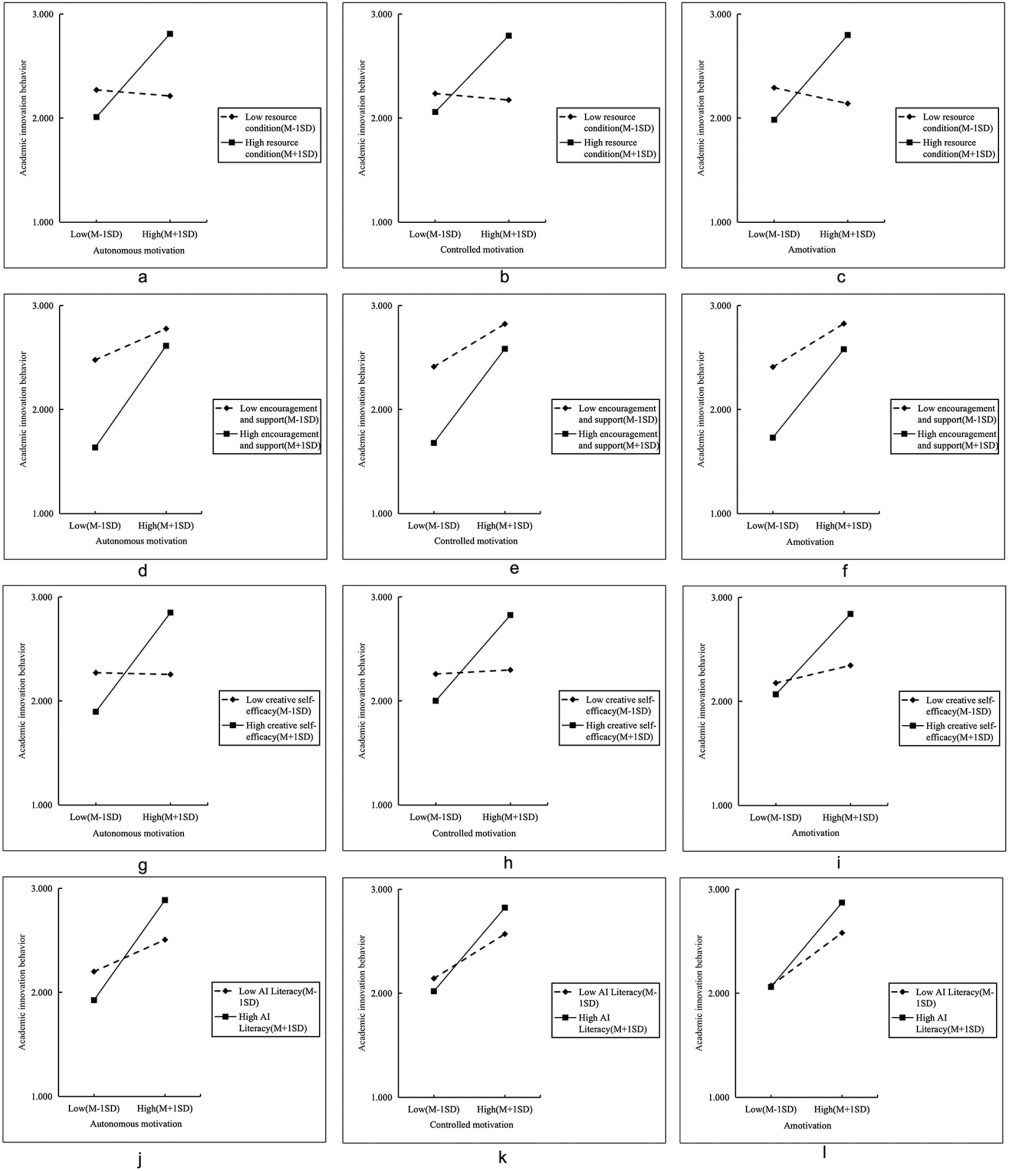
Simple slope.

Moderation model was tested by PROCESS Macro. To examine the moderation, the predictor variable (autonomous motivation, controlled motivation or amotivation), the moderating variable (creative self-efficacy, AI literacy, resource condition, or encouragement and support), and academic innovation behavior were added to the model. Table 5 showed the results of moderation analyses. Fig 2 indicated simple slope.

**Table 5:** Results of moderation effect.

Construct	Interaction term	*b*	SE	Result
Resource condition	Autonomous motivation × resource condition	0.299***	0.041	Supported
	Controlled motivation × resource condition	0.303***	0.042	Supported
	Amotivation × resource condition	0.356***	0.042	Supported
Encouragement and support	Autonomous motivation × encouragement and support	0.186***	0.039	Supported
	Controlled motivation × encouragement and support	0.147***	0.042	Supported
	Amotivation × encouragement and support	0.126***	0.042	Supported
Creative self-efficacy	Autonomous motivation × creative self-efficacy	0.400***	0.046	Supported
	Controlled motivation × creative self-efficacy	0.354***	0.052,	Supported
	Amotivation × creative self-efficacy	0.265***	0.052	Supported
AI Literacy	Autonomous motivation × AI Literacy	0.227***	0.053	Supported
	Controlled motivation × AI Literacy	0.142***	0.055	Supported
	Amotivation × AI Literacy	0.111***	0.053	Supported

Firstly, we examined the moderating role of RC. A significant positive interaction effect emerged between AM and RC on AIB (*b* = 0.299, SE = 0.041, *p* < 0.001), supported H4a. Specifically, as shown in Fig 2(a), the slope for high RC (*b* = 0.468, SE = 0.046, *p* < 0.001) is steeper than for low RC (*b* = −0.034, SE = 0.063, *p* = 0.590), indicating that when RC is high, academic doctoral students with AM have more positive AIB. In addition, the result revealed that RC had a promoting effect on the positive between CM and AIB (*b* = 0.303, SE = 0.042, *p* < 0.001), supported H4b. Specifically, as Fig 2(b) shows, the size of this promoting effect effect was stronger at a higher levels of RC (*b* = 0.468, SE = 0.051, *p* < 0.001), suggesting that RC may be particularly important for strengthening the positive impact of CM on AIB. Finally, the result revealed that RC had a damping effect on the negative relationship between M and AIB (*b* = 0.356, SE = 0.042, *p* < 0.001), supported H4c. As Fig 2(c) shows, simple slope tests revealed the positive relationship between M and AIB was weaker at low RC (b = −0.094, SE = 0.066, p = 0.152) compared to low RC (b = 0.501, SE = 0.048, p < 0.001).

Regarding ES, this result showed displayed a promoting moderation effect on positive relationship between AM (*b* = 0.186, SE = 0.039, *p* < 0.001), CM (*b* = 0.147, SE = 0.042, *p* < 0.001) and AIB, supporting H5a and H5b. Meanwhile, ES had a damping effect on the negative relationship between M and AIB (*b* = 0.126, SE = 0.042, *p* < 0.001), supporting H5c. As shown in Fig 2(d), Fig 2(e) and Fig 2(f), this positive relationship between AM, CM, M and AIB was stronger than at higher levels of ES than at lower levels.

Regarding CS, this result showed displayed a promoting moderation effect on positive relationship between AM (*b* = 0.400, SE = 0.046, *p* < 0.001), CM (*b* = 0.354, SE = 0.052, *p* < 0.001) and AIB. Moreover, CS had a damping effect on the negative relationship between M (*b* = 0.265, SE = 0.052, *p* < 0.001) and AIB. Thus, H6a, H6b and H6c were supported. As shown in Fig 2(g), Fig 2(h) and Fig 2(i), this promoting and damping effect were stronger than at higher levels of CS than at lower levels.

In addition, the result showed that AIL strengthened the positive relationship AM (*b* = 0.227, SE = 0.053, *p* < 0.001), CM (*b* = 0.142, SE = 0.055, *p* < 0.001), and AIB. Furthermore, AIL had a damping effect on the negative relationship between M (*b* = 0.111, SE = 0.053, *p* < 0.001) and AIB. These results showed that H7a, H7b and H7c were supported. As shown in Fig 2(j), Fig 2(k) and Fig 2(l), this promoting effect and damping were stronger than at higher levels of AIL than at lower levels.

## Discussion

Academic doctoral students serve as the main force in knowledge production and a reservoir of future scientists. Studying their academic innovation behavior plays a crucial role in advancing the fundamental theories required for the nation to overcome “chokepoint” technologies, thereby helping the country maintain a sustainable reserve of talent in the face of intense international scientific and technological competition.In recent years, GenAI technology has developed rapidly and become widely adopted across various fields. Research has shown that this technology plays a significant role in driving academic innovation [69].This study, grounded in SDT and the MOA framework, investigated the predictive effects of different types of academic motivation on doctoral students’ academic innovation behavior, alongside the moderating roles of resource conditions, encouragement and support, creative self-efficacy, and AI literacy. Our findings extend existing research while offering new understandings of the motivational mechanisms underlying academic innovation in the GenAI era.

### Direct effects of motivation on innovation behavior

Our results confirm that autonomous academic motivation significantly and positively predicts academic innovation behavior. This aligns with the core tenet of SDT that intrinsically motivated individuals exhibit greater persistence, curiosity, and willingness to engage in cognitively demanding tasks [70]. As Tadić, Vujčić et al. pointed out, intrinsic academic interest can encourage individuals to demonstrate greater initiative and innovation in their academic work [23]. Academic innovation is inherently characterized by high uncertainty, high cognitive load, and long-term returns, and requires sustained mental effort [71].Doctoral students who experience genuine interest and enjoyment in their research are more likely to invest sustained effort in novel and risky inquiries, even when facing high uncertainty and heavy workloads [72,73].This finding resonates with recent research by Yang [74], who demonstrated that AI-assisted language learning environments can foster self-regulation and autonomy, thereby enhancing learners’ proactive behaviors. Extending this logic to the doctoral research context, our study suggests that autonomous motivation serves as an internal engine that drives academic innovation, particularly when academic doctoral students perceive their work as meaningful and self-endorsed.

Furthermore, our results reveal that controlled motivation significantly and positively predicts academic innovation behavior—a finding that appears paradoxical within classical SDT postulations which traditionally posit external regulation as detrimental to creativity [75].However, we argue that in the high-stakes ecosystem of doctoral education—characterized by time-bound graduation mandates, publication pressures, and competitive job markets [76]—controlled motivation does not operate as a mere coercive force. When academic doctoral students perceive external requirements (e.g., degree completion) as personally relevant challenges rather than decontextualized threats, these regulations can be partially internalized, supplying the goal clarity and sustained effort necessary for navigating multi-year, high-uncertainty innovative projects. Our result nuances the findings of Derakhshan & Park (2026), while they found that AI adoption pressures stimulate adaptation via coping resources [77], our study specifies that in doctoral research, this adaptation is contingent upon the perceived instrumentality of external rules. In survival-oriented academic contexts, the detrimental effect of controlled motivation on innovation is moderated by institutional reward salience and short-term goal proximity [78].

Finally, research has found that a lack of motivation for academic innovation among academic doctoral students has a significant negative impact on their innovative behaviors. Specifically, the absence of academic innovation motivation reflects the thwarting of doctoral students’ basic psychological needs for autonomy and competence in the research process. According to SDT, when these intrinsic needs are frustrated, individuals’ regulatory orientation shifts toward an external perceived locus of causality, which fundamentally undermines their cognitive flexibility, tolerance for uncertainty, and propensity for intellectual risk-taking which are indispensable for generating novel scholarly contributions.The empirical findings of this study confirm that doctoral students who lack research motivation find it difficult to maintain deep, long-term engagement in research work [79]. Building on this, the present study draws on SDT to analyze the underlying mechanisms, thereby deepening our understanding of this issue. Previous research based on SDT has confirmed that intrinsic motivation can enhance graduate students’ commitment to research, thereby positively predicting their innovative capacity [80].Moreover, doctoral students with intrinsic motivation traits tend to exhibit greater perseverance in research, higher satisfaction with their research, and superior academic performance [81]. However, most existing studies have focused on positive pathways, emphasizing the promotional effects of motivation on research innovation.This study adds to the understanding of negative mechanisms in this field by revealing the inhibitory effect of lack of motivation on doctoral students’ innovative behavior. Previous studies have largely attributed this phenomenon of innovation constraints directly to behavioral-level deficiencies, whereas this study clarifies the core psychological mechanism: a lack of motivation depletes academic doctoral students’ sustained cognitive engagement, making it difficult for them to maintain key research behaviors such as iterative reasoning and repeated trial and error which constitute the very essence of academic innovation.

### Moderating roles of resources, support, self-efficacy, and AI literacy

Beyond the direct effects, our moderation analyses reveal critical boundary conditions that either amplify or attenuate the motivation-innovation relationships. These moderators can be grouped into opportunity (resource conditions, encouragement and support) and ability (creative self-efficacy, AI literacy) factors, and they jointly shape how motivational energy is transformed into innovative action.

First, resource conditions-including funding, laboratory equipment, and literature access—significantly amplified the positive effects of both autonomous and controlled motivation, while buffering the negative impact of amotivation. By integrating SDT with the MOA framework, this study provides a multifaceted theoretical explanation for the aforementioned findings. From the perspective of SDT, adequate resources satisfy the basic needs for competence and relatedness, enabling doctoral students to conduct experiments without fear of failure [82]. This aligns with the ecological perspective of Yang and Derakhshan [83], which emphasizes how green spaces shape teachers’ AI literacy and, in turn, influence their innovative practices. However, this study also yields new findings that differ from classical theories: research resources can equally empower controlled motivation. Classical self-determination theory posits that interventions and stimuli from the external environment undermine the purity and sustainability of an individual’s intrinsic motivation [84]. However, in high-pressure, high-risk doctoral research settings, research resources actually serve to “reduce cognitive load,” effectively lowering the implementation costs of innovative exploration and research execution. With the support of comprehensive resources, even when students’ research behaviors stem from external rules and requirements, they can successfully transform extrinsic research goals into substantive research outcomes. For unmotivated individuals, abundant resources—by lowering the costs of innovative implementation and providing feedback on competence—enable them to sustain academic innovation even in the absence of intrinsic motivation, thereby effectively mitigating the negative effects of such a lack of motivation.

Second, encouragement and support served as a powerful positive moderator. Consistent with existing research, support of supervisors for students’ innovation positively moderates the relationship between autonomous motivation and academic engagement [80]. When supervisors actively endorse and reward innovative attempts, doctoral students perceive a supportive climate that reduces the perceived risk of failure, thereby strengthening their willingness to pursue novel directions. Our results show that encouragement is effective for those with controlled motivation, perhaps. According to classical SDT, controlled motivation is inherently unlikely to drive stable, positive. This study offers an academic-context-based explanation for this paradox. Specifically, faculty encouragement and organizational support help doctoral students transform external demands into goals aligned with their personal identity, compensating for deficiencies in intrinsic motivation and sustaining continuous progress in their research. This suggests that effective encouragement and support can facilitate the transition from external regulation to internal integration, enabling controlled motivation to serve a functional purpose in specific contexts.At the same time, this study further reveals the buffering effect of encouragement and support on amotivation, thereby expanding the theoretical boundaries of SDT. Encouragement and support not only serve as instrumental conditions but also alleviate academic doctoral students’ tendency to avoid research uncertainties by fostering a sense of psychological safety. For individuals with insufficient commitment or a negative mindset, positive incentives can break through negative cognitions, stimulate their willingness to actively experiment and explore, and reduce the psychological burden associated with the consequences of failure, thereby effectively curbing the continued loss of motivation.

Third, creative self-efficacy significantly moderates the relationship between motivation and academic innovation behavior. Specifically, creative self-efficacy amplifies the positive effects of both autonomous and controlled motivation on academic doctoral students’ academic innovation, while buffering the detrimental impact of amotivation. We argue that creative self-efficacy functions differentially across motivational types. For students with existing motivation, high creative self-efficacysustains goal commitment and buffers anxiety, maximizing the utility of their drive when facing research setbacks. Critically, for amotivated students, creative self-efficacy does not amplify (as there is no drive to amplify) but rather plays a compensatory initiation role that buffers the negative amotivation-innovation relationship. Specifically, high creative self-efficacy lowers the psychological threshold for task initiation by providing a minimal sense of “I can do it” independent of any internal or external reason to act, and enables amotivated students to reframe research uncertainty as manageable trial-and-error rather than threat, thereby re-engaging them in minimal-viable innovation practices. This compensatory interpretation aligns with Li et al.’s (2026) finding that enhancing self-efficacy effectively buffers the adverse effects of external pressure on academic outcomes [85].Recent evidence further supports this view, for example, Özbay and Köse (2025) found that self-efficacy mediated the effects of various motivational regulations on academic behaviors, implying that efficacy beliefs can reduce procrastination even when motivation is low [86]. Accordingly, within the MOA analytical framework, we define creative self-efficacy as a mechanism for screening differential abilities. When individuals possess both intrinsic and extrinsic motivation, creative self-efficacy amplifies the driving force of motivation; if intrinsic motivation is insufficient, creative self-efficacy can compensatorily drive individuals to take action.

Fourth, and most notably, AI literacy emerged as a significant moderator, strengthening the motivation–innovation link and weakening the amotivation-innovation negative relationship. While existing research predominantly conceptualizes AI literacy as a direct predictor of job performance or a mediator of engagement [87,88], our findings substantially extend this literature by identifying its moderating and compensatory functions. Specifically, we demonstrated that AI literacy is not only an independent driver but also plays a moderating role. For motivated students, AI literacy amplifies exploration by lowering experimental trial-and-error costs. Crucially, for amotivated students, high AI literacy serves as a substitute for internal drive—by providing algorithmic assistance that reduces cognitive load and fear of failure, it effectively breaks the “avoidance loop”. Our finding suggests that technological ability (AI literacy) can structurally compensate for motivational deficits even when psychological self-beliefs remain low, thus offering a novel intervention pathway for academic support systems.

## Conclusion

First, academic doctoral students’ academic innovation behavior is driven by a dual-motivation pathway. Unlike the conclusions drawn by classical theories in conventional educational settings, this study focuses on high-pressure academic training environments in chemistry to conduct an empirical analysis, confirming that both intrinsic and extrinsic motivation serve as effective driving forces for academic doctoral students’ academic innovation. These two types of motivation do not exist in a zero-sum, opposing relationship as traditionally perceived; rather, they can coexist and synergize in specific research contexts, jointly exerting a positive driving effect. However, when academic doctoral students are in a prolonged state of motivational inactivation—where their basic psychological needs remain unmet—their cognitive flexibility, tolerance for research uncertainty, and willingness to engage in academic exploration will decline markedly, thereby significantly inhibiting the emergence of academic innovation. This conclusion indicates that the mechanism of academic doctoral students’ academic motivation exhibits distinct contextual specificity and cannot be simply explained by applying general educational theories.

Second, external resources and individual traits form a multidimensional moderating ecosystem that serves the dual functions of reinforcing innovation and buffering risks. This study confirms that core moderating variables—such as research resource reserves, external research support, and creative self-efficacy—do not merely exert a single, linear, positive effect on academic innovation. When academic doctoral students possess sufficient internal research motivation, various external resources and positive individual traits can further empower innovative behavior, accelerating academic exploration and the production of research outcomes; when doctoral students have weak research motivation or are in a low-motivation state, high-quality external conditions and favorable individual traits can serve as a safety net. By lowering the barriers to implementing research innovation, fostering a safe and stable psychological environment for research, and supporting the practical implementation of academic exploration, these factors help maintain doctoral students’ basic academic innovation vitality and effectively compensate for the loss of innovation resulting from a lack of internal motivation. This also confirms that a favorable external research environment and positive individual traits can effectively compensate for the shortcomings in innovation caused by insufficient motivation.

Third, individual AI literacy is a key moderating factor for academic doctoral students’ academic innovation in the era of GenAI. This study innovatively finds that individual AI literacy serves as the core mediating link and key moderating factor connecting research motivation and academic innovation behavior. Academic doctoral students with solid AI literacy can effectively overcome various barriers in research, optimize the research exploration process, and enhance the efficiency of academic trial-and-error, innovative exploration, and the translation of research outcomes. Consequently, they significantly strengthen the empowering effect of research motivation on academic innovation behavior and optimize the efficiency of translating motivation into innovation. This conclusion fully demonstrates the important role that the research literacy system in the GenAI era plays in shaping current academic research paradigms and the logic of knowledge production.

## Implications

### Theoretical implications

Based on an integrated perspective combining SDT and the MOA framework, this study, through empirical testing, has made some theoretical contributions, effectively advancing the application and development of the relevant theoretical framework in the high-pressure research environment faced by academic doctoral students.

First, we expand the scope of self-determination theory and reveal the context-specific nature of motivation. Classical self-determination theory adheres to a binary opposition in which “autonomous motivation is positive and controlled motivation is negative” [89], and is primarily applicable to conventional educational settings. This study focuses on the high-pressure research training environment for academic doctoral students and revises the theoretical assertions of this theory. The study found that in a high-pressure environment characterized by academic evaluations, research publications, and industry competition, both intrinsic and extrinsic motivation can positively drive academic innovation among doctoral students, while only motivational disengagement significantly inhibits innovative behavior. This result confirms that motivational efficacy is significantly context-dependent. In high-pressure situations, individuals can reframe external pressure as a growth challenge, enabling extrinsic motivation to become an effective support for research innovation [81,90]. Motivational disengagement leads to a lack of basic psychological needs, resulting in reduced cognitive flexibility and lower tolerance for research challenges, which ultimately hinders innovative exploration. This study extends the explanatory scope of SDT to high-pressure academic contexts, refines the mechanisms of motivational transformation under stressful conditions, and effectively broadens the theory’s applicability.

Second, by integrating the MOA framework, this study reconstructs the moderating mechanisms underlying the motivation-to-behavior transition. By combining SDT with the MOA analytical framework, this study sheds light on the “black box” in existing research—which tends to emphasize motivational outcomes while neglecting the transition process. Unlike traditional research, which views moderating variables as having a linear effect, this study confirms that research resources, external support, creative self-efficacy, and AI literacy function as composite moderating variables that serve both empowering and buffering roles. Positive research resources and external support can reduce the costs of innovation and foster a research environment that tolerates mistakes [91–93], while individual self-efficacy and AI literacy can enhance the efficiency of scientific research exploration [94]. This research challenges the one-sided view in traditional theory that regards the external environment as a negative interference, confirming that internal and external supporting factors can work synergistically to construct a scientific research ecosystem. This ecosystem not only strengthens the innovation-driving role of positive motivation but also cushions the negative impact of motivational inactivation, maintaining the vitality of basic innovation when individual motivation is low. It reaffirms the central supporting role of the environment and individual traits in the transformation of motivation.

Third, clarify the central role of artificial intelligence literacy and fill the gap in academic innovation research on AI. Existing studies largely view artificial intelligence literacy as a foundational research skill and a predictor of performance [13], overlooking its deeper value in the mechanism of motivational transmission. This study addresses this research gap by clarifying that AI literacy serves as a key moderating variable linking individual motivation to academic innovation, rather than merely a research tool. Strong AI literacy can break through technical barriers in scientific research and optimize the effectiveness and transmission pathways of motivation on innovative behavior. This finding confirms the transformation in the logic of academic production in the intelligent era: AI has been deeply integrated into the entire process of scientific research—from cognition and exploration to output—and has become a core ecological resource. By incorporating technological ecological elements into the framework of motivation research, this study provides a new theoretical basis for constructing a model of academic innovation dynamics in the context of GenAI.

### Practical implications

Based on the findings of this study, the following insights are offered to key stakeholders involved in the training of research-oriented doctoral students:

First, universities and training institutions need to establish an institutional environment that balances “developmental pressure” with “supportive resources”. Universities need not deliberately avoid reasonable pressures related to graduation and publication; rather, they should appropriately transform rigid assessment requirements into “growth-oriented challenges”. Specifically, while setting clear output standards, they should provide adequate research funding, laboratory equipment, and opportunities for academic exchange, and establish routine psychological support and academic safety-net mechanisms. Even if academic doctoral students temporarily experience a dip in motivation, high-quality resource support and a safe environment can help them maintain basic research vitality and prevent them from becoming completely “inactive”. At the same time, AI literacy training should be strengthened, with a focus on reinforcing academic autonomy. Academic doctoral students should be guided to carefully evaluate the utility of AI tools throughout the entire research process, strictly adhere to the boundaries of data ethics, and remain vigilant against the risks of technological misuse [95], thereby ensuring that AI technology truly serves the production of original knowledge rather than undermining the independence of academic judgment.

Second, supervisors of academic doctoral students need to accurately assess their students’ motivation levels and focus on preventing the risk of “motivation burnout.” Supervisors should avoid a “one-size-fits-all” approach to motivation. For students with strong intrinsic motivation, supervisors should grant them ample academic autonomy; for students with prominent extrinsic motivation, supervisors should help them break down external goals into clear, actionable step-by-step plans, transforming pressure into concrete cognitive anchors. At the same time, supervisors need to shift their focus from “whether students are working hard” to “whether students have lost their curiosity and desire to explore.” Once tendencies toward cognitive avoidance are detected, they should promptly provide emotional support and intervention.

Finally, academic doctoral students must proactively reframe their perception of stress and actively build their AI literacy. They should acknowledge the objective reality of high-pressure environments and view graduation requirements and publication targets as “action scaffolds” for advancing complex innovative projects—rather than purely external constraints—thereby harnessing the positive effects of controlled motivation. At the same time, they should recognize that AI literacy is not merely a tool for enhancing efficiency, but also a lever for reshaping research paradigms. On the one hand, they should proactively integrate AI into processes such as literature mining, experimental design, and data analysis to reduce the cost of trial and error and expand the boundaries of exploration; on the other hand, they must maintain a habit of critical verification and strictly adhere to the bottom lines of academic integrity and data security [96], and continuously reflect on and update one’s own AI usage strategies, thereby ensuring that human researchers retain their agency and independence even as they are empowered by technology.

### Limitations and future research directions

Several limitations warrant consideration in future research. First, this study primarily employs a questionnaire survey method, and since the data are derived from self-reports collected at a single point in time, common method bias may be present. Since academic innovation behavior is a dynamic and evolving process, cross-sectional data struggle to capture the dynamic impact of changing motivations on innovation behavior over time and cannot strictly infer the causal direction between variables. Although we have statistically controlled for academic year factors, resource conditions, encouragement and support, creative self-efficacy, and AI literacy may play different roles at various stages of doctoral training. At the same time, we acknowledge the possibility of reverse causality: higher levels of academic innovation behavior may lead to reports of higher creative self-efficacy. To address these issues, future research could employ multi-time-point or longitudinal tracking surveys to determine the direction of causality, more accurately reveal the dynamic evolution of how motivation influences innovation behavior, and assess the prominence of resource conditions, encouragement and support, creative self-efficacy, and AI literacy at each stage.

Second, the mechanisms underlying the relationships among the variables have not been thoroughly explored. This study only verified the moderating effects of resource conditions, encouragement and support, and creative self-efficacy on AI literacy, but did not delve deeply into the underlying mechanisms linking these variables. For example, do AI literacy, resource conditions, and encouraging support indirectly influence the relationship between motivation and academic innovation behavior by affecting creative self-efficacy? Aside from creative self-efficacy, are there other mediating or moderating variables that influence the relationship between motivation and academic innovation behavior? These questions remain unanswered, resulting in insufficient theoretical depth and explanatory power regarding the underlying mechanisms. Future research could further investigate and clarify the hierarchical relationships among these variables. Concurrently, more potential mediating or moderating variables—such as psychological resilience, organizational innovation climate, and academic identity—could be introduced to construct a more comprehensive theoretical model and provide a deeper analysis of the pathways influencing academic innovation behavior. Furthermore, targeted studies could be conducted by integrating characteristics such as gender, discipline, and training type to explore the unique influencing factors and mechanisms of doctoral students’ academic innovation behavior, thereby providing a theoretical basis for differentiated innovation training and support strategies.

Finally，this study primarily employed a questionnaire survey method to collect data, relying heavily on respondents’ subjective perceptions. Such self-reported data may be influenced by social desirability bias, potentially leading to skewed results. Additionally, as the questionnaire survey method primarily consists of closed-ended questions, it can only capture superficial correlations and intuitive outcomes between variables; it cannot delve into the underlying logic behind these variables, such as the differences in the origins of autonomous and controlled motivation, or the specific situational factors contributing to the formation of a state of amotivation. Future research will supplement this approach with qualitative methods such as in-depth interviews and case studies to conduct a thorough investigation, uncovering the underlying logic, situational factors, and individual experiences underlying the relationship between different types of motivation and academic innovation behavior.
