# Correction: Exploring the influencing factors of academic doctoral students’ academic innovation behavior in the context of generative artificial intelligence: Self-determination theory and motivation-opportunity-ability perspectives

**DOI:** 10.1371/journal.pone.0357707

**Published:** 2026-09-02

**Authors:** Yanqin Liu, Miao Zhang, Yuwen Peng

The Data Availability statement for this article is incorrect. The correct statement is: Due to ethical restrictions and participant privacy concerns, the datasets generated and/or analyzed during the current study are not publicly available. Access to the data can be requested from the Medical Ethics Review Committee Office of Xizang University at 3197523122@qq.com or send a formal request to the committee office’s postal address: Medical Ethics Review Committee Office, Xizang University, No. 26 Niangre Road, Chengguan District, Lhasa, Xizang Autonomous Region, 850000, China. Data requests will be reviewed by the committee to ensure compliance with applicable privacy and consent requirements.

## References

[1] Liu Y, Zhang M, Peng Y. Exploring the influencing factors of academic doctoral students’ academic innovation behavior in the context of generative artificial intelligence: Self-determination theory and motivation-opportunity-ability perspectives. PLoS One. 2026;21(8):e0355186. doi: 10.1371/journal.pone.0355186 42560956 PMC13446694

